# Autocatalytic flow chemistry

**DOI:** 10.1038/s41598-023-36360-5

**Published:** 2023-06-06

**Authors:** Csenge Galanics, Virág Sintár, István Szalai

**Affiliations:** Institute of Chemistry, Eötvös Loránd University, Budapest, 1117 Hungary

**Keywords:** Chemistry, Physical chemistry, Reaction kinetics and dynamics

## Abstract

Autocatalysis is a crucial process of nonequilibrium self-organization in nature and is assumed to play a role in the origin of life. The essential dynamical phenomena of an autocatalytic reaction network are bistability and the development of propagating front when combined with diffusion. The presence of bulk fluid motion may widen the range of emerging behavior in those systems. Many aspects of the dynamics of autocatalytic reactions in a continuous flow have already been studied, especially the shape and dynamics of the chemical front and the influence of the chemical reactions on hydrodynamic instabilities. This paper aims to provide experimental evidence of bistability and related dynamical phenomena, such as excitability and oscillations in autocatalytic reactions performed in a tubular flow reactor, where the flow is laminar and advection is the dominating transport process. We show that the linear residence time ramp may result in the simultaneous appearance of different dynamic states along the length of the pipe. Therefore, long tubular reactors offer a unique opportunity to quickly explore the dynamics of reaction networks. These findings enhance our understanding of nonlinear flow chemistry and its role in natural pattern formation.

## Introduction

Autocatalysis appears in reactions of small molecules, macromolecules, and the supramolecular level^1,2^, and it is at the heart of life’s chemistry^3^. Replication and exponential growth caused by autocatalytic networks are essential natural self-organization processes from the molecular to the population level. The universal characteristics of the autocatalytic processes inherently connect these distant areas. Beyond the temporal dynamics, compelling spatiotemporal patterns may appear when transport processes are combined with autocatalytic networks. Both diffusion, a molecular-level transport process, and bulk fluid motion, advection can play a constructive role in developing these phenomena.Turing patterns are an emblematic example where autocatalysis combined with negative feedback and differential diffusion produces self-organized patterns^4^.

**Figure 1:**
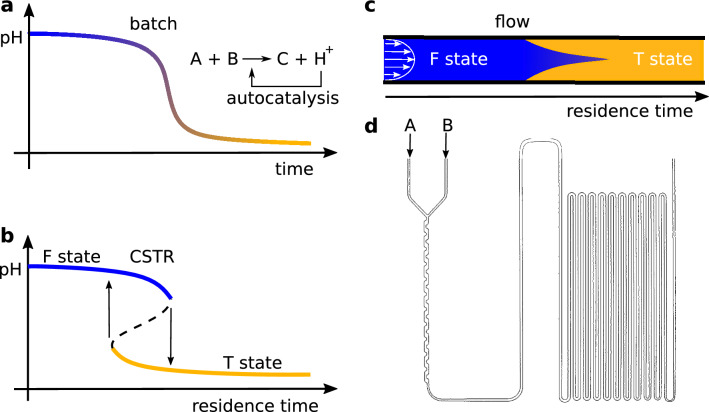
Relevant properties of pH-autocatalytic networks: sigmoidal pH *vs.* time curve in batch (**a**), bistability in a CSTR (**b**), and stretched front in adverse laminar flow (**c**). Sketch of the flow reactor with a pipe diameter of 1 mm used in the experiments (**d**).

**Figure 2:**
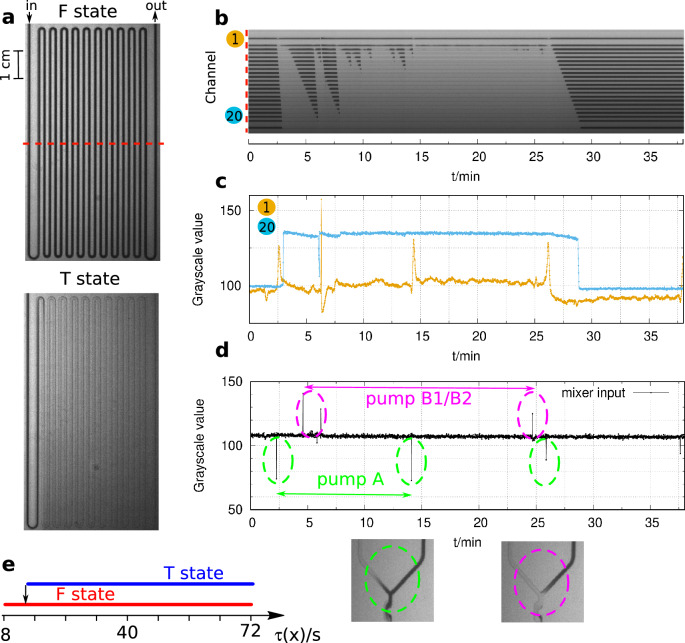
Bistability in the flow reactor in the chlorite–tetrathionate reaction (**a**). Dark color corresponds to high and light color to low pH. The space-time plot represents the spatiotemporal dynamics in the middle of the reactor (**b**). Representative local dynamics is shown in the middle of channel 1 and 20 (**c**). The stability of the flow is followed at the input of the mixer unit, where a spike denotes a backflow (**d**). Diagram of the stability of the states (F and T) along the tube (**e**). Experimental conditions: $[\hbox {ClO}_{2}^-]_0$ = 19 mM, $[\hbox {S}_{4}\hbox {O}_{6}^{2-}]_0$ = 5 mM, $\left[ {{\text{H}}_{2} {\text{SO}}_{4} } \right]_{0}$ = 0.6 mM, $v_0$ = 96 mL/h, *u*= 3.4 cm/s, T= 25 $^{\circ }\hbox {C}$.

**Figure 3:**
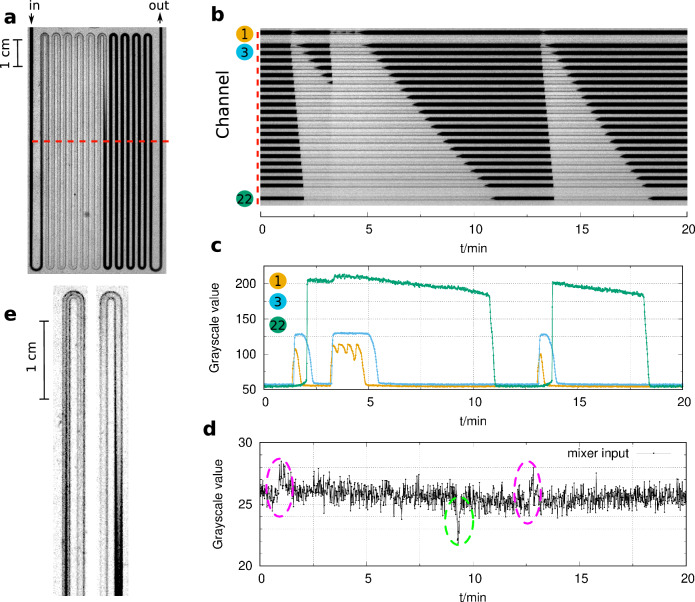
Excitabilty in the flow reactor in the bromate–sulfite reaction (**a**). Dark color corresponds to high and light color to low pH. The space-time plot represents the spatiotemporal dynamics in the middle of the reactor (**b**). Representative local dynamics is shown in the middle of channel 1, 3, and 22 (**c**). The stability of the flow is followed at the input of the mixer unit, where a spike denotes a backflow (**d**). Representative profiles of the two different fronts (**e**). Experimental conditions: $[\hbox {BrO}_{3}^{-}]_0$ = 30 mM, $[\hbox {SO}_{3}^{2-}]_0$ = 60 mM, $[\hbox {H}_{2}\hbox {SO}_{4}]_0$ = 5 mM, $v_0$ = 40 mL/h, *u*= 1.4 cm/s, T= 25 $^{\circ }\hbox {C}$.

**Figure 4:**
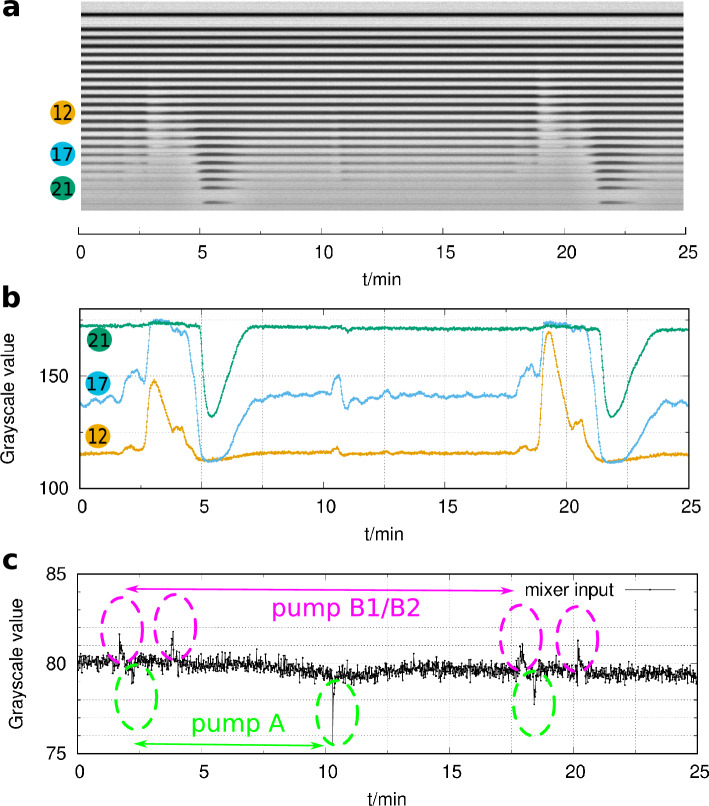
Excitabilty in the flow reactor in the iodate–sulfite reaction. Dark color corresponds to high and light color to low pH. The space-time plot represents the spatiotemporal dynamics in the middle of the reactor (**a**). Representative local dynamics is shown in the middle of channel 12, 17, and 21 (**b**). The stability of the flow is followed at the input of the mixer unit, where a spike denotes a backflow (**c**). Experimental conditions: $[\hbox {IO}_{3}^{-}]_0$ = 15 mM, $[\hbox {SO}_{3}^{2-}]_0$ = 60 mM, $[\hbox {H}_{2}\hbox {SO}_{4}]_0$ = 20 mM, $v_0$ = 140 mL/h, *u*= 4.9 cm/s, T= 25 $^{\circ }\hbox {C}$.

The general kinetics and dynamics of autocatalytic chemical networks have been explored under many conditions^1,2,5^. In a batch reactor the sigmoidal concentration versus time curve (Fig. 1a) and in a continous-stirred-tank reactor (CSTR) the bistability (Fig. 1b) are the fingerprint of these systems. In a CSTR, low residence time favors a state where the extent of the overall reaction is low (F state). At high residence time, the reaction is almost complete (T state). The stability range of these two stationary states overlaps in a certain residence time range, where the two states coexist. An autocatalytic chemical system that shows bistability can be purposefully extended by adding a negative feedback reaction to show excitability and oscillations in a CSTR^6,7^.

A straightforward case where transport processes come into play is the development of a sharp traveling chemical front in an autocatalytic reaction-diffusion system that travels with a constant speed determined by the kinetic and diffusional properties^8,9^. The propagating flat front may become unstable in differential diffusion and give rise to a cellular front^10^. Extended systems with negative chemical feedback are ready to produce an enormous diversity of reaction-diffusion patterns^4,7,11^.

The presence of bulk flow adds new sources of instabilities to the previously discussed chemical and diffusional ones due to the mutual interaction between nonlinear chemistry and the flow properties, e.g., flow rate, density, and viscosity^12–15^. Laminar flow significantly affects the rate of the reaction and the shape of the reaction-diffusion front, depending on the direction of the flow^16–19^. Flow is called supportive when the flow’s direction and the chemical reaction front’s propagation are the same. Supportive flows produce large reaction front velocities that exceed the sum of the velocity of a planar front in the absence of flow and the average flow velocity. Adverse flow, where the direction of the flow and the propagation of the chemical front is opposite, stretches the front in the direction of the flow and may result in a cusp shape front (Fig. 1c). In this case, the front can be even stationary. Recently, numerical simulations predicted that for strong enough flow velocities, the autocatalytic fronts might become oscillatory^20^.

Here, we present an experimental study of four different autocatalytic networks in a commercial tubular flow reactor with a diameter of 1 mm and length 216 cm (Fig. 1d). At the applied conditions, the flow is laminar (Re$\sim$10–140), and the role of diffusive mixing is small as the Peclét number is high ($>10^3$). We aimed to explore the effect of the linear residence time ramp alongside the reactor on the dynamics of the autocatalytic reactions. The operation of long laminar flow reactors can be described as a series of well-mixed reactors (tanks-in-series model), where a number of equally sized ideal CSTRs are connected^21^. Therefore, long laminar flow reactors can easily collect time-series data^22^. Following this idea, we anticipate that bistability and the related dynamical phenomena which appear in a CSTR could be observed in a flow reactor. We used hydrogen ion, and hydroxide ion autocatalytic reactions in the experiments, which produce a significant pH drop, and the variation of pH can be followed by indicators.

We choose the chlorite–tetrathionate (CT) reaction because it produces bistability in a CSTR over a wide range of the control parameters^23^. The kinetics of the reaction is rather complex^24,25^, but its hydrogen ion autocatalytic nature is often described by the following equation:

R1 $$\begin{aligned} \hbox {7ClO}_{2}{}^{-} + \hbox {2S}_{4}\hbox {O}_{6}{}^{2-} +\hbox {6H}_{2}\hbox {O} \longrightarrow \hbox {7Cl}^{-} + \hbox {8SO}_4{}^{2-} + 12\hbox {H}^{+} \end{aligned}$$

1 $$\begin{aligned} v_{1}=k_{1}[\hbox {ClO}_{2}{}^{-}][\hbox {S}_{4}\hbox {O}_{6}{}^{2-}][\hbox {H}^{+}]^2 \end{aligned}$$

The reaction rate is quite slow in alkaline conditions but in slightly acidic conditions, the reaction starts immediately (see Supplementary Fig. S1 online). We did not find reports in the literature where adding a hydrogen ion-consuming reaction to the CT systems would result in oscillations in a CSTR. Therefore, the CT reaction can be treated as a system that produces solely bistability in a CSTR. In the domain of bistability, the pH of the F state is above 9, and that of the T state is below 2.

The bromate–sulfite (BS) and the iodate–sulfite (IS) reactions are the prototypes of Landolt reactions. We selected these reactions because the presence of a kinetic negative feedback process quickly turns them to oscillatory in a CSTR. During the BS reaction in a batch reactor, setting the initial pH to around 7 in the non-buffered medium, after a slow pH decrease of roughly 5–7 min, the pH of the mixture suddenly drops to around 2–3 (see Supplementary Fig. S2 and S3 online)^26^ .The following steps can summarize the mechanism of the reaction:^27^

R2 $$\begin{aligned} \hbox {SO}_{3}{}^{2-} + \hbox {H}^{+} \rightleftharpoons \hbox {HSO}_{3}{}^{-} \end{aligned}$$

R3 $$\begin{aligned} \hbox {HSO}_{3}{}^{-} + \hbox {H}^{+} \rightleftharpoons \hbox {H}_{2}\hbox {SO}_{3} \end{aligned}$$

R4 $$\begin{aligned} \hbox {BrO}_{3}{}^{-} + \hbox {3HSO}_{3}{}^{-} \longrightarrow \hbox {Br}^{-} + \hbox {3SO}_{4}{}^{2-} + \hbox {3H}^{+} \end{aligned}$$

R5 $$\begin{aligned} \hbox {BrO}_{3}{}^{-} + \hbox {3H}_{2}\hbox {SO}_{3} \longrightarrow \hbox {Br}^{-} + \hbox {3SO}_{4}{}^{2-} + \hbox {6H}^{+} \end{aligned}$$

R6 $$\begin{aligned} \hbox {BrO}_{3}{}^{-} + \hbox {6H}\hbox {SO}_3{}^{-} \longrightarrow \hbox {Br}^{-} + \hbox {3S}_{2}\hbox {O}_{6}{}^{2-} + \hbox {3H}_{2}\hbox {O} \end{aligned}$$

Reactions (R2)–(R5) are responsible for the formation of autocatalysis. Reaction (R6) is a slow reaction consuming hydrogen ions. As a result of this, in addition to bistability, oscillation can also develop in a CSTR under suitable conditions^27^. In the domain of bistability, the pH of the F state is 6–7, and that of the T state is between 2 and 3. The BS reaction can be combined with many other hydrogen ion scavenging reactions^28^. These extended systems are well suited for the investigation of temporal and spatial phenomena^29,30^.

The IS reaction is autocatalytic for hydrogen ions and iodide ions^31,32^. The reaction mechanism can be described using the following three reaction equations:

R7 $$\begin{aligned} \hbox {IO}_{3}{}^{-} + \hbox {3HSO}_{3}{}^{-} \longrightarrow \hbox {I}^{-} + \hbox {3SO}_{4}{}^{2-} + \hbox {3H}^{+} \end{aligned}$$

R8 $$\begin{aligned} \hbox {IO}_{3}{}^{-} + \hbox {5I}^{-} + \hbox {6H}^{+} \longrightarrow \hbox {3I}_{2} + \hbox {3H}_{2}\hbox {O} \end{aligned}$$

R9 $$\begin{aligned} \hbox {I}_{2} + \hbox {HSO}_{3}{}^{-} + \hbox {H}_{2}\hbox {O} \longrightarrow \hbox {2I}^{-} + \hbox {SO}_{4}{}^{2-} + \hbox {3H}^{+} \end{aligned}$$

Performing the IS reaction in an unbuffered medium and setting the initial pH to around 8 in a batch reactor, after a slow pH decrease of approximately 5–7 minutes, the pH of the mixture suddenly drops to around 4 (see Supplementary Fig. S4 online). After it, the reaction (R8) may cause a slow increase in pH if iodate ions are in excess. In a CSTR, the IS reaction shows bistability^31^, and oscillation was observed only in extended versions. In the domain of bistability, the pH of the F state is 6–8, and that of the T state is between 2 and 3.

The fourth selected reaction is the formaldehyde–sulfite (FS) reaction. The FS reaction differs from the previous ones in two respects. One of its peculiarities is that the autocatalytic species in this reaction is the hydroxide ion. Perhaps more importantly, this is not an inorganic redox chemical but a fundamentally organic chemical reaction. To describe the reaction, Taylor and coworkers proposed the following mechanism^33–35^:

R10 $$\begin{aligned} \hbox {CH}_{2}\hbox {(OH)}_{2} \rightleftharpoons \hbox {CH}_{2}\hbox {O} + \hbox {H}_{2}\hbox {O} \end{aligned}$$

R11 $$\begin{aligned} \hbox {HSO}_{3}{}^{-} \rightleftharpoons \hbox {SO}_{3}{}^{2-} + \hbox {H}^{+} \end{aligned}$$

R12 $$\begin{aligned} \hbox {CH}_{2}\hbox {O} + \hbox {SO}_{3}{}^{2-} \longrightarrow \hbox {CH}_{2}\hbox {(O}^{-}\hbox {)SO}_{3}{}^{-} \end{aligned}$$

R13 $$\begin{aligned} \hbox {CH}_{2}\hbox {(O}^{-}\hbox {)SO}_{3}{}^{-} + \hbox {H}^{+} \rightleftharpoons \hbox {CH}_{2}\hbox {(OH)SO}_{3}{}^{-} \end{aligned}$$

R14 $$\begin{aligned} \hbox {H}_{2}\hbox {O} \rightleftharpoons \hbox {H}^{+} + \hbox {OH}^{-} \end{aligned}$$

R15 $$\begin{aligned} \hbox {CH}_{2}\hbox {O} + \hbox {HSO}_{3}{}^{-} \longrightarrow \hbox {CH}_{2}\hbox {(OH)SO}_{3}{}^{-} \end{aligned}$$

R16 $$\begin{aligned} \hbox {CH}_{2}\hbox {(OH)}_{2} + \hbox {SO}_{3}{}^{2-} \longrightarrow \hbox {CH}_{2}\hbox {(O}^{-}\hbox {)SO}_{3}{}^{-} + \hbox {H}_{2}\hbox {O} \end{aligned}$$

R17 $$\begin{aligned} \hbox {CH}_{2}\hbox {(OH)}_2 + \hbox {HSO}_{3}{}^{-} \longrightarrow \hbox {CH}_{2}\hbox {(OH)SO}_{3}{}^{-} + \hbox {H}_{2}\hbox {O} \end{aligned}$$

In a batch reactor, setting the initial pH between 6–7, after a short period (10–30 s), the pH of the mixture suddenly rises to 10–12 (see Supplementary Fig. S5 online). In a CSTR, bistability, and oscillations were also observed^33–35^. In this reaction, a pH of 6–8 characterizes the F state, and the pH of the T state is about 12.

The experiments with this complementary set of these four autocatalytic networks, which have similar sigmoidal-type batch kinetic and bistable CSTR dynamics but naturally have some individual aspects (e.g., the BS and FS reactions include negative kinetic feedbacks), are suitable for exploring the fundamental behavior of autocatalytic chemistry in a laminar flow reactor.

## Results and discussion

The first step of our experiments was to find the conditions at which the transition from the unreacted (F state) to the reacted (T state) of the autocatalytic reaction can be seen in the reactor, as the residence time and the extent of the reaction changes along the tube. Typically, two parameters were changed: one parameter influencing the induction time of the given autocatalytic reaction (e.g., the acid feed concentration), and the other the flow rate determining the average total residence time of the flow reactor. The goal was to match the length of the induction period that can be measured in the batch reactor and the average total residence time of the flow reactor.

**Figure 5:**
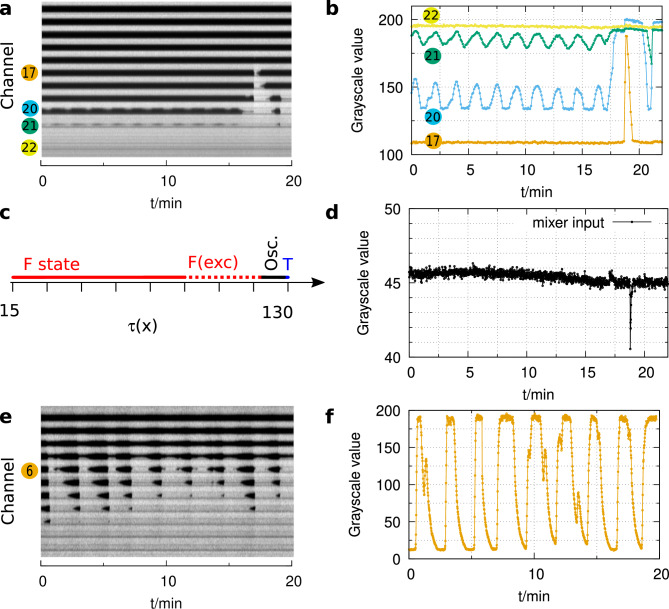
Oscillations in the flow reactor in the bromate–sulfite reaction (**a**). Dark color corresponds to high and light color to low pH. The space-time plot represents the spatiotemporal dynamics in the middle of the reactor. Representative local dynamics is shown in channel 17, 20,21, and 22 (**b**). Diagram of the stability of the states along the tube (**c**). The stability of the flow is followed at the input of the mixer unit, where a spike denotes a backflow (**d**). Oscillations observed using single syringe pumps: space-time plot (**e**) and local dynamics in the middle of channel 6 (**f**). Experimental conditions: $[\hbox {BrO}_{3}^{-}]_0$ = 330 mM, $[\hbox {SO}_{3}^{2-}]_0$ = 60 mM, $[\hbox {H}_{2}\hbox {SO}_{4}]_0$ = 5 mM, $v_0$ = 52 mL/h, *u*= 1.8 cm/s, T= 25 $^{\circ }\hbox {C}$.

**Figure 6:**
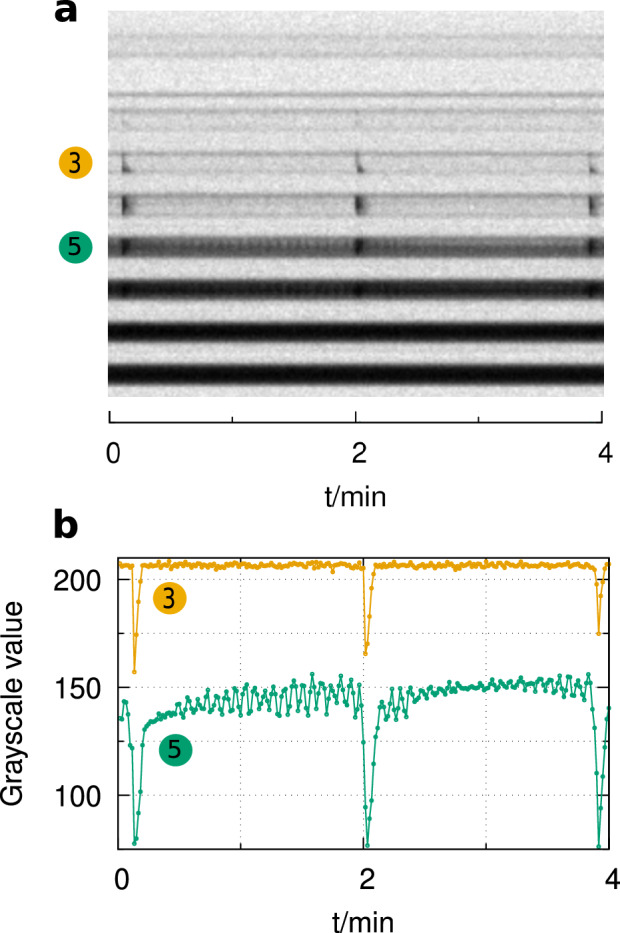
Dynamics of formaldehyde–sulfite reaction in flow: space-time plot (**a**) and local dynamics in the middle of channel 3 and 5 (**b**). Experimental conditions: $[\hbox {CH}_{2}\hbox {O}]_0$ = 0.4 mM, $[\hbox {SO}_{3}^{2-}]_0$ = 0.01 mM, $[\hbox {S}_{2}\hbox {O}_{5}^{2-}]_0$ = 0.05 mM, $v_0$ = 300 mL/h, *u*= 10.6 cm/s, T= 25 $^{\circ }\hbox {C}$.

**Figure 7:**
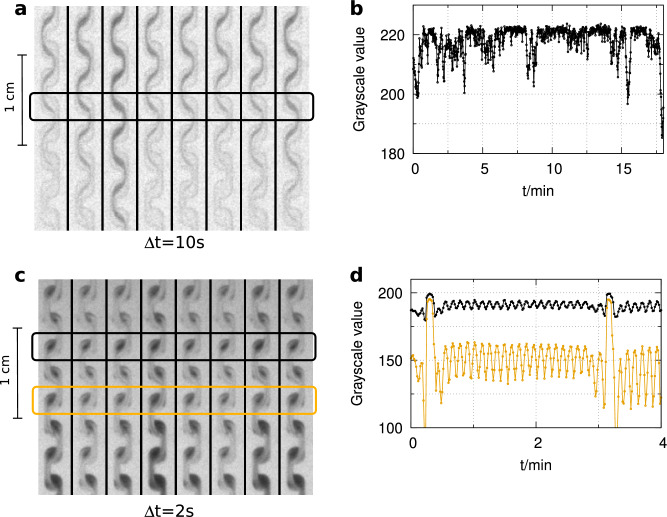
Oscillations in the bromate–sulfite (**a** and **b**) and in the formaldehyde-sulfite (**c** and **d**) reactions in flow reactor designed for better mixing. Experimental conditions: $[\hbox {BrO}_{3}^{-}]_0$ = 330 mM, $[\hbox {SO}_{3}^{2-}]_0$ = 60 mM, $[\hbox {H}_{2}\hbox {SO}_{4}]_0$ = 5.0 mM, $v_0$ = 32 mL/h, *u*= 1.1 cm/s, and $[\hbox {CH}_{2}\hbox {O}]_0$ = 0.4 mM, $[\hbox {SO}_{3}^{2-}]_0$ = 0.01 mM, $[\hbox {S}_{2}\hbox {O}_{5}^{2-}]_0$ = 0.05 mM, $v_0$ = 400 mL/h, *u*= 14.1 cm/s, T= 25 $^{\circ }\hbox {C}$.

### Bistability

According to the previous studies, in an adverse flow, a stationary front may develop in an autocatalytic reactions^16–19^. This front connects the F and T states of the reactions. In an open system, the appearance of such a front is associated with bistability. Experiments with CT reaction are suitable to explore the development of bistability in a laminar flow reactor as the two stationary states of the reaction are stable over broad parameters, such as residence time.

The experiments were started by slowing down the flow rate to reach the T (pH$\sim 2$, light color) state at the outflow but keeping the F (pH$>6$, dark color) state in the central part of the reactor (Fig. 2a). At this condition, the F state is stable until perturbations in the flow occur.

The operation of the applied double-piston chromatography pumps caused the perturbations. In these pumps pumping action is provided by the two alternating pistons: one piston draws in the solution while the other expels solution. The flow stability could be followed at the entrance point of the reactor where the two flows are mixed (Fig. 2d). When the direction of the movement of the cylinders in the pumps changes, a small backflow is recorded at the mixing of the input flows. The backflows appear as sharp spikes in Fig. 2d. The pump delivered the chlorite solution (pump A) operated two times faster than the two other pumps (pump B1 and B2), which delivered the sulfuric acid and the tetrathionate solutions. These latter flows were combined to get the same flow rate at the two inputs of the mixing unit (Fig. 1d). Therefore, the perturbation period caused by pump A is half of the perturbations caused by the other pumps.

As a result of the perturbations, at around 2 min, the reaction switched to the T state (Fig. 2a) in a large tube domain. This new spatial state is also stable. The back transition requires another perturbation of around 25 minutes. The space-time plot (Fig. 2b) shows that the transition from the F to the T state is faster than the backward one. The F to T transition starts from the outlet part and propagates at $\sim 6.5$ cm/s along the tube, with a faster rate than the actual flow rate of 3.4 cm/s. The propagation rate of the transition from the T state to the F is much slower, $\sim 1.0$ cm/s. The F state always remains stable (yellow curve in Fig. 2c) in the first segment of the channel as the short local residence time at this tube segment supports the F state. In the other part of the reactor, e.g., in segment 20, appropriate perturbations result in transitions between the F and T states (blue curve in Fig. 2c). By comparing Fig. 2c and d, it is clear that transitions only occur due to the perturbation caused by the pumps. The local residence time ($\tau$) increases linearly along the length of the tube, as $\tau (x)=l(x)/u$, where *u* is the flow rate and *l*(*x*) is the distance from the mixing point. Therefore, we can conclude that over a large domain of residence time (tube length), the F and the T states may coexist (Fig. 2e), that is bistability.

### Excitability

The Landolt-type BS and IS reactions also show bistability in a CSTR^26,31^. Still, we did not find clear evidence for bistability in our experiments in the tubular flow reactor. We started the experiments with the BS reaction at a high flow rate where the entire reactor is in the F (high pH) state. When the flow rate was decreased, the acidic state appeared in the middle segment of the tube (Fig. 3a) and propagated with a rate of 5 cm/s. This rate is significantly faster than the flow rate, which is 1.4 cm/s (Fig. 3b). The appearance of the T state was triggered by a flow disturbance caused by the pumps, shown in Fig. 3c and d. In these experiments, the slight color difference between the input feed solutions made registering the backflows caused by the pumps difficult. The T state was unstable at these conditions, and recovery to the F state started spontaneously. Supplementary movie 1 shows this dynamic behavior. The propagation of the F state at the expense of the T state is slower. The rate is about 0.7 cm/s. The two types of fronts are strongly stretched, as shown in Fig. 3e. It is interesting to notice that at the first segment of the tube, the perturbation triggers transient oscillations, as seen in Fig. 3c (yellow curve).

Similar phenomena were observed in the IS reaction. We could not observe bistability, but the perturbations caused transient changes in the local state. At the first half of the tube, the F state is stable, and the perturbations do not cause any observable changes (Fig. 4a). In the second half of the reactor, from segment 10, a gradual change in the indicator’s color was observed, indicating a relatively smooth change from the F to the T state. When a perturbation occurs at 2.5 minutes, the F state temporally switches to the T state between segments 11 and 15 (Fig. 4b and c). This transition propagated with a rate of 2 cm/s, which is lower than the actual flow rate (4.9 cm/s).

A noisy intermediate color state is formed in the next segment of the reactor. In this region,e.g., in segment 17, a perturbation caused a large excursion that started with a change to the F state. However, the F state is unstable. After a sudden transition to the T state, the noisy intermediate color state finally resettled. At the last part of the reactor (e.g., segment 21), where the T state is the stable state, the perturbations cause a transient change to the F state. Important to notice that the perturbations caused by the pump delivering the iodate solution have no significant effect on the state of the reacting mixture in channel segments 12 and 21 (Fig. 4b and c). However, they resulted in transient damped oscillations in the intermediate region (in segment 17), as seen after the perturbation at 10 min.

In our experiments with the BS and IS reactions, we could not observe bistability at the applied conditions. However, tiny flow disturbances can trigger dramatic transient changes in the state of the reaction mixture at some regions of the flow reactor. These events appear locally and propagate through a limited region of the reactor. We assume that these phenomena correspond to the excitability of the system. Excitability is a general phenomenon in a bistable system combined with negative feedback. In the flow reactor, the interplay of the hydrogen ion consuming steps (reaction (R6) and (R9)) and flow of fresh reactants can provide the necessary negative feedback. Although we could not define a threshold for the stimuli that result in an action, the IS reaction experiments demonstrate that only one type of flow disturbance is effective.

### Oscillations

In a simple autocatalytic network, the development of oscillations is unexpected as it requires negative feedback. Only the BS and FS reactions among the selected systems show oscillations in a CSTR due to their kinetic complexity.

In the BS system, the negative feedback is produced by reaction (R6)^27^, which is effective at high bromate excess. The experiments with the ratio of $[\hbox {BrO}_{3}^{-}]_0/[\hbox {SO}_{3}^{2-}]_0=5.5$ were started at a high flow rate, where all along the tube, the reactive mixture is at the F state. When the flow rate was decreased, different dynamics were observed at different positions along the tube (Fig. 5a and b). The F state was stable in the first part of the reactor, e.g., between segments 1 and 16. In the next part, between segments 17 and 19, the F state was also stable, but in this region, it is excitable. The region excitability is followed by a domain of oscillations (Fig. 5a–d) along the tube. The perturbations caused by the pumps stop the oscillations for a while, but then they restart (see Supplementary Fig. S9 online). The period of these oscillations is about 1.6 min, which is surprisingly short compared to the previous observations in CSTR, where the typical period was in the order of hours^27^. The short-period oscillations can not be solely induced by the kinetic feedback of the reaction (R6). We explain that the interplay of the flow and the kinetic feedback of the reaction (R6) results in the observed oscillations.

To check the role of the applied pumps in the appearance of oscillations, we performed experiments with two alternative pump types. Single syringe pumps avoid the perturbations caused by the alterations of the movement of the cylinders in chromatography pumps, but the syringes’ volume limits the experiment’s time length. The observed space-time plot (Fig. 5e) and the local dynamics (Fig. 5f) demonstrate that the appearance of oscillations is not connected to the flow disturbances caused by the pumps in the previous experiments. Supplementary movie 2 shows the oscillatory behavior. We also found oscillations using peristaltic pumps (see Supplementary Fig. S10 online), but according to our observations, this pumping method is unsuitable, probably due to the significant flow noise^36^.

The chemistry of the FS reaction is quite different from the systems we discussed before. The autocatalytic species is a hydroxide ion, and it does not involve any strong oxidant like the CT, BS, and IS reactions. In a CSTR the FS retactions shows bistability between a slightly alkaline (pH=7–8) F state and an alkaline (pH$\sim$11) T state and short period ($\sim$30 s) oscillations^33^.

The experiments were started at the F state (light color in Fig. 6) as before. An appropriate decrease in the flow rate resulted in the appearance of the T state (dark color in Fig. 6) at the final segment of the tube. In between excitability, e.g., in segment 3, and oscillations (e.g., in segment 5) were observed. The period of the oscillations is about 5 s. The short period indicates that the contribution of both the negative chemical feedback and the flow plays a role in the periodic behavior.

We performed additional experiments in a specific zigzag shape microreactor (see Supplementary Fig. S6 online) designed for mixing sensitive reactions. The specific geometry of the channels created dead volumes where the state of the mixture differed from that of the main flow. Figure 7 shows snapshots of the oscillations recorded in the BS (Fig. 7a and b) and in the FS (Fig. 7c, and d) reactions in that microreactor and Supplementary movie 3 shows the oscillatory behavior observed in the FS reaction. The autocatalytic reactions draw out the channel geometry’s significant effect on the reaction’s local state. The appearance of the periodic behavior in the zigzag shape microreactor, where the laminar nature of the flow is intentionally disturbed, shows the flow-supported oscillatory phenomenon’s robustness.

## Conclusion

Microreactors’ commercial availability opens a convenient way to explore nonlinear phenomena in laminar flow. We used a set of four autocatalytic reactions, which have complementary properties, to gain general insight. Not surprisingly, the autocatalytic reactions show sensitivity to flow disturbances. In our experiments, the regular small backflows caused by the pumps provided perturbations that we could use to test the stability of the stationary states of the system. However, the sensitivity of autocatalytic reactions to flow disturbances makes it challenging to perform long-lasting experiments. According to the literature, using HPLC pumps, backpressure regulators, and mass flow meters might help avoid this problem^37,38^. Definately, monitoring the flow rate’s stability is critical when the dynamics within the flow reactor is studied. As an approximation, the operation of a long tubular reactor with the laminar flow can be described as a series of coupled CSTRs^21^. We anticipated that the bistability and other related phenomena, which could be observed in a CSTR, could also be developed in a tubular flow reactor.

Our findings indicate that bistability, excitabilty, and oscillations can be easily observed in laminar flow reactors by appropriately tuning the timescales of the reactions and that of the flow. In contrast to a CSTR in a tubular flow reactor, the gradients of the local residence time significantly affect the dynamics. The tube length (*L*) is an essential factor, as it determines the total residence time of the flow reactor, $\tau =L/u$. The gradient of the local residence time ($\tau (x)$) along the tube is determined by 1/*u*. In order to match $\tau$ to the timescale of the reaction in a longer tube higher flow rate must be used. Therefore, a longer tube allows us to set a smaller gradient of $\tau (x)$, which helps stabilize the different states along the tube.

Our observations agree with the general picture of the dynamics of bistable systems with negative feedback, which was analyzed by Boissonade and De Kepper^6^ and Guckenheimer^39^ in detail by using a mathematically tractable model. They have shown that as the strength of the negative feedback increases, the domain of bistability decreases, and new dynamical phenomena, e.g., excitability and oscillations develop. We assume that the adverse flow, where the direction flow is opposite to the natural direction of the autocatalytic front, provides negative feedback. This flow-induced feedback is readily combined with the kinetics ones and results in the appearance of excitability and oscillations. However, many open questions exist, e.g., how does the stretched front geometry affect the observed behavior, or how do the flow and the chemical negative feedback interplay?

Flow chemistry is a fast-developing field that offers a broad range of applications and naturally opens a way for the nonequilibrium synthesis of materials with kinetically trapped structures^37,40^. The insights gained from this study may assist in efficiently exploring the dynamics of autocatalytic chemical and biochemical networks in flow microreactors. Besides the known advantages of these reactors, as shown here, they allow quick exploration of the different dynamic states in a single experiment. The constructive role of the flow presented here might also help to build oscillatory supramolecular systems, which have limited known experimental evidence but are assumed to have great significance as models of minimal life forms^41–43^.

## Methods

The experiments were performed in flow reactors with a diameter of 1 mm, which has a shorter premixer section and a more extended reaction area. Supplementary Figure S6 shows the mixing unit and the two different reactors with tubular and zigzag shapes, the latter of which ensures better mixing. The reactors are made of borosilicate glass (manufacturer: Little Things Factory). The length of the tubular reactor is 216.6 cm. The reactants are fed into the two inlet branches of the premixer. We used such flow rates where in the premixer unit, the progress of the reaction is low. The applied volume flow rate range was $v_0$=40–400 mL/h. As the pipe cross-section was $A=7.85\times 10^{ -3}$cm$^2$, the flow rate, *u*=$v_0/A$, range was 5.1$\times 10^{3}$–$10^{4}$ cm/h. Taking the kinematic viscosity of aqueous solutions as approximately $10^{-6}$ m$^2$/s, the typical Reynolds number for the flow is 14–140. Therefore the flow in our experiments is laminar. The estimated value of the Peclét number, taking the diffusion coefficient as $2\times 10^{-9}$ m$^2$/s, is $7\times 10^{3}-10^{4}$. Therefore the role of diffusion is small compared to flow, and the use of the mixing unit was important since mixing by diffusion is also negligible. Based on the length of the tubular reactor (216.6 cm) and the approximate length of the mixing unit (25 cm), the average total residence time was between $\tau =$16 and 160 s. In the case of the zigzag shape reactor, the average total residence time is approximately half of this.

A schematic drawing of the entire experimental equipment is shown in Supplementary Figure S7 and S8. We used three different pump types. Most of the experiments were performed by chromatography pumps (Pharmacia Biotech Pump P-500). The three different stock solutions were flowed by three pumps. One pump was connected directly to the mixing unit and supplied the oxidizing agent (bromate, iodate, or chlorite). The solutions delivered by the other two pumps (sulfite/tetrathionate and sulfuric acid) were delivered to the mixing unit after a premixing. The pump’s flow rate, which delivered the oxidizing agent, was always twice that of the other two pumps. The reactor units were immersed in a thermostatic bath. In the case of the FS reaction, only two pumps were used with equal flow rates. The reactor was illuminated from below with an LED lamp. The black and white images were recorded using a digital camera (ImagingSource) and a 600 nm 10 nm wide optical bandpass filter (Edmund Optics). The images were processed by ImageJ.^44^

To test the effect of the pumps, we also used two other pump types: two 50 mL syringe pumps and a four-channel peristaltic pump. When the single syringe pumps were used, one pump contained the oxidizing agent, and the other a mixture of sulfite and sulfuric acid solutions. The two pumps operated at the same flow rate. The disadvantage of this type of pump is that the solution in the syringes is only sufficient for limited-time experiments. The oxidizing agent was delivered on two branches when the four-channel peristaltic pump was used (Gilson Minipuls 3). The sulfite/tetrathionate and the sulfuric acid solutions were on the one-one branch.

The stock solutions used in the experiments are summarized in Supplementary Table 1. We used deionized water to prepare the solutions. To follow the pH change, bromocresol green ($10^{-4}$ M, transition range pH$=$3.8–5.4, yellowish-green-blue, light absorption maximum at pH$=$5.4 615–618 nm) and in the case of the FS reaction thymol blue ($5\times 10^{-4}$ M, transition range pH=7.8–9.5 yellowish-green-blue, light absorption maximum at pH=5.4 594–598-618  nm) indicator was used. In the recorded black and white images, the dark color corresponds to pH>5.5 (bromocresol green) and pH>9.5 (thymol blue). The behavior of the reactions in a batch reactor was investigated using a glass electrode (Hanna) in a 25 mL thermostated reactor.

## Supplementary Information

Supplementary Information 1.

Supplementary Information 2.

Supplementary Information 3.

Supplementary Information 4.

## Data Availability

The datasets used and/or analysed during the current study available from the corresponding author on reasonable request.
