# Supplementary material for: Autocatalytic flow chemistry
Source: Sci Rep. 2023 Jun 6;13:9211. doi: 10.1038/s41598-023-36360-5 (PMC10244366; doi:10.1038/s41598-023-36360-5)
Supplement: Supplementary file 1 — Supplementary Information 1. [file 41598_2023_36360_MOESM1_ESM.pdf]

Autocatalytic flow chemistry

Supplementary information

Csenge Galanics[1], Virág Sintár[1] and István Szalai[1*]

[1*]Institute Of Chemistry, Eötvös L. University, Pázmány P. s. 1/A, Budapest, 1117, Hungary.

*Corresponding author(s). E-mail(s): istvan.szalai@ttk.elte.hu; Contributing authors: galakcsenge@gmail.com; sintarvirag01@gmail.com;

- Figure S1: pH *vs.* time curves in the chlorite-tetrathionate reaction in batch.
- Figure S2: pH *vs.* time curve in the bromate–sulfite reaction in batch.
- Figure S3: pH *vs.* time curve in the bromate–sulfite reaction in batch.
- Figure S4: pH *vs.* time curve in the iodate–sulfite reaction in batch.
- Figure S5: pH *vs.* time curve in the formaldehyde–sulfite reaction in batch.
- Figure S6: Reactor units.
- Figure S7: Experimental setup with three chromatography pumps.
- Figure S8: Alternative pumping methods.
- Figure S9: Oscillations in the flow reactor in the bromate–sulfite reaction in channel 21.
- Figure S10: Oscillations in the flow reactor in the bromate–sulfite reaction observed by using a peristaltic pump.
- Table 1: Stock solutions used in the experiment
- Supplementary movie 1: Excitability in a flow reactor (BS reaction)
- Supplementary movie 2: Oscillations in a flow reactor (BS reaction)
- Supplementary movie 3: Oscillations in a flow reactor (FS reaction)

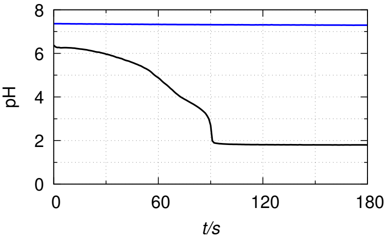

Fig. S1 pH *vs.* time curves in the chlorite-tetrathionate reaction in batch, starting at pH=7.4 (blue) and pH=6.2 (black). Experimenetal conditions: $[ClO_2^{-}]_0 = 19\,mM$, $[S_4O_6^{2-}]_0 = 5\,mM$, T= 25 ℃.

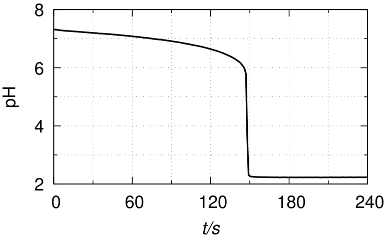

Fig. S2 pH *vs.* time curve in the bromate–sulfite reaction in batch. Experimenetal conditions: $[BrO_3^{-}]_0 = 30\,mM$, $[SO_3^{2-}]_0 = 60\,mM$, $[H_2SO_4]_0 = 8\,mM$, T= 25 ℃.

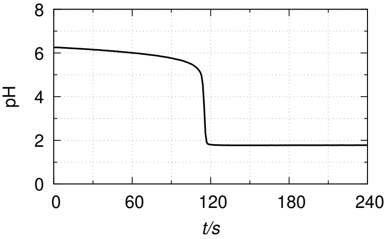

Fig. S3 pH *vs.* time curve in the bromate–sulfite reaction in batch. Experimenetal conditions: $[BrO_3^{-}]_0 = 330\,mM$, $[SO_3^{2-}]_0 = 60\,mM$, $[H_2SO_4]_0 = 5\,mM$, T= 25 ℃.

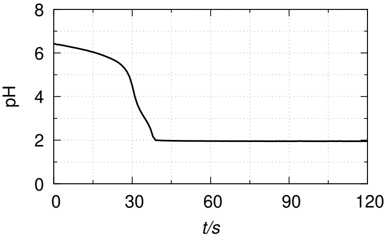

Fig. S4 pH *vs.* time curve in the iodate–sulfite reaction in batch. Experimenetal conditions: $[IO_3^{-}]_0 = 15\,mM$, $[SO_3^{2-}]_0 = 60\,mM$, $[H_2SO_4]_0 = 20\,mM$, T= 25 ℃.

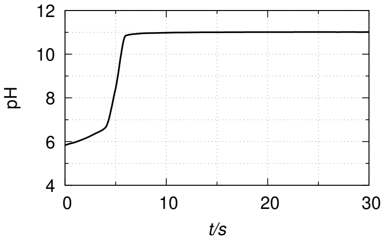

Fig. S5 pH *vs.* time curve in the formaldehyde–sulfite reaction in batch. Experimenetal conditions: $[CH_2O]_0 = 0{,}4$ mM, $[{SO_3}^{2-}]_0 = 0{,}01$ mM, $[{S_2O_5}^{2-}]_0 = 0{,}05$ mM, T= 25 °C.

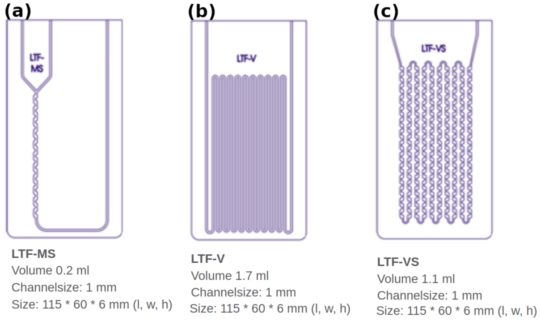

Fig. S6 Reactor units: premixer (a), tubular reactor (b), and zigzag shape reactor (c). The reactors are the product of Little Things Factory.

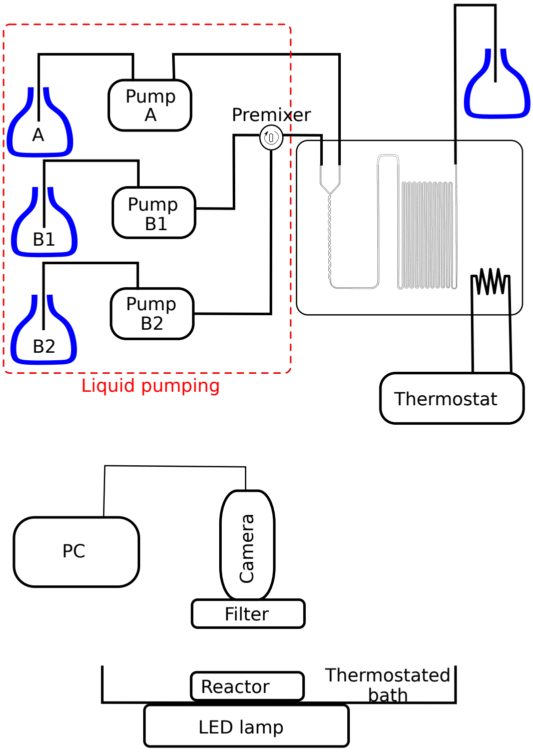

Fig. S7 Experimental setup with three chromatography pumps.

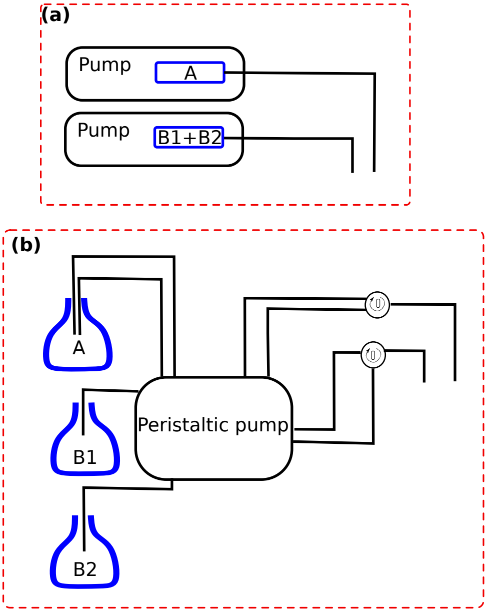

Fig. S8 Alternative pumping methods: single syringe pumps (a), a peristaltic pump (c).

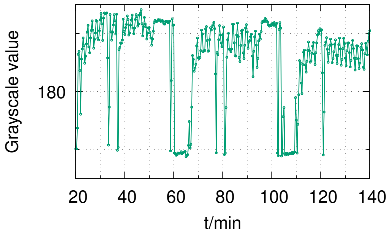

Fig. S9 Oscillations in the flow reactor in the bromate–sulfite reaction in channel 21. Experimental conditions: $[BrO_3{}^-]_0 = 330\,mM$, $[SO_3{}^{2-}]_0 = 60\,mM$, $[H_2SO_4]_0 = 5\,mM$, $v_0 = 52\,mL/h$, T= 25 °C.

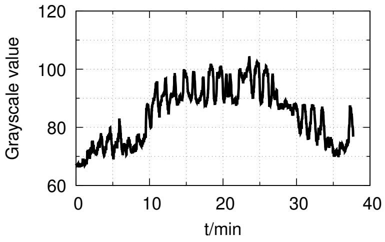

Fig. S10 Oscillations in the flow reactor in the bromate–sulfite reaction observed by using a peristaltic pump. Experimenetal conditions:$[BrO_3{}^-]_0 = 330\,mM$, $[SO_3{}^{2-}]_0 = 60\,mM$, $[H_2SO_4]_0 = 5\,mM$, $v_0 = 50\,mL/h$, T= 25 °C.

Table 1 Stock solutions used in the experiments

Stock solution	Concentration
Bromate–sulfite reaction	
$NaBrO_3$ (Sigma, puriss. p.a.)	0.66 M
Na_2SO_3 (Sigma, ACS reagent)	0.24 M
H_2SO_4 (VWR, titrinorm)	0.02 M
Bromate–sulfite reaction	
$NaBrO_3$ (Sigma, puriss. p.a.)	0.06 M
Na_2SO_3 (Sigma, ACS reagent)	0.24 M
H_2SO_4 (VWR, titrinorm)	0.02 M
Iodate–sulfite reaction	
KIO_3 (Sigma, ACS reagent)	0.30 M
Na_2SO_3 (Sigma, ACS reagent)	0.24 M
H_2SO_4 (VWR, titrinorm)	0.08 M
Chlorite–tetrathionate reaction	
$NaClO_2$ (Sigma, puriss. p.a.))	0.038 M
$K_2S_4O_6$ (Sigma, $\geq$98.0%)	0.02 M
H_2SO_4 (VWR, titrinorm)	0.0024 M
Formaldehyde–sulfite reaction	
CH_2O (Sigma, ACS reagent)	0.8 M
Na_2SO_3 (Sigma, ACS reagent)/$Na_2S_2O_5$ (Sigma, ReagentPlus)	0.02 M/0.1 M
